# First Principles of Physics or Natural Philosophy

**Published:** 1859-03

**Authors:** 


Art. VIII.—*First Principles of Physics or Natural Philosophy.* By Benjamin Silliman, Jr., M.A., M.D., Professor of General and Applied Chemistry in Yale College. 12mo., pp. 720. Philadelphia: Henry C. Peck & Theodore Bliss. 1859.

Among the many works which are issued from the press, it is pleasant to find occasionally a book which fulfills the wants of the student. The majority of the text-books on natural philosophy may be divided into two classes: those which have been simplified to adapt them to the comprehension of "babes and sucklings," and those which are so exclusively mathematical that one glance at their abstruse formulæ is quite sufficient to satisfy any ordinary reader.

But here we have a work free from these peculiarities, which is equally valuable as a text-book and as a book of reference.

The subjects are judiciously arranged, the chief principles of the science being given in large type, while the illustrations and matters of secondary importance are thrown into smaller type. In all the details of the science the work is a model of fullness and precision, and its facts are brought down to the day of publication. Our attention has been particularly called to the able manner in which the author has systematized the facts in regard to the imponderables—heat, light, and electricity. The sections on optics and acoustics will be read with special interest by mem bers of the medical profession.

It is always a great satisfaction to consult a scientific work by an au thor on whose facts we can rely, and this book affords a capital illustration of the great advantage—so rarely met with in these days of paste and scissors—of an author's possessing an intimate and critical knowledge of the subjects upon which he writes.

Our thanks are due to Professor Silliman for this valuable addition to our scientific literature, and the publishers deserve great credit for the liberal manner in which the work is illustrated, as well as for printing it on capital paper and issuing it in an elegant and substantial binding.

